# Clinical placement education during the coronavirus disease 2019 pandemic shapes new nurses: A qualitative study

**DOI:** 10.1016/j.ijnsa.2023.100145

**Published:** 2023-07-17

**Authors:** Monika Ravik, Etty Ragnhild Nilsen, Marianne Wighus, Randi Garang Mofossbakke, Gro Gade Haanes

**Affiliations:** Department of Nursing and Health Sciences, Faculty of Health and Social Sciences, University of South-Eastern Norway, Post-box 235, 3603, Kongsberg, Norway

**Keywords:** Coronavirus disease 2019 pandemic, Clinical placement experiences, Newly-qualified nurses, Nursing education, Transition, Transition-related challenges

## Abstract

**Background:**

Many newly qualified nurses experience transition challenges because they feel unprepared for the reality of the nursing profession owing to the theory-practice gap. Transition challenges amongst newly qualified nurses have profound consequences for the nursing profession and patient care. A detailed and nuanced understanding of the complexity in transition-related challenges during extraordinary conditions like the coronavirus disease 2019 pandemic is needed for newly qualified nurses to remain in the nursing profession.

**Objective:**

This study explored the experience of newly qualified nurses entering the nursing profession who had their clinical placement education missed, shortened, or substituted since they had to serve as health care assistants during the coronavirus disease 2019 pandemic.

**Design:**

An exploratory and descriptive study design was used.

**Settings:**

Workplaces for newly qualified nurses.

**Participants:**

A purposive sample of 10 newly qualified Norwegian nurses working in various clinical placement nursing settings were included.

**Methods:**

Data were collected in April and May 2022 via individual interviews conducted via Zoom. Thematic analysis was applied to identify themes. Triangulation was employed to ensure trustworthiness.

**Findings:**

Three major themes emerged: relational aspects of nursing, practical aspects of nursing, and inadequacies in the pedagogical plan of teaching and learning. The themes indicate that the limited or suspended clinical placement education during the pandemic affected the competence of newly qualified nurses.

**Conclusions:**

During the coronavirus disease 2019 pandemic, clinical placement education for student nurses was limited or suspended owing to safety concerns. The short clinical placement durations affected the competence of newly qualified nurses, as they lacked exposure to real-world patient care scenarios as in non-pandemic times. Furthermore, our findings indicate that newly qualified nurses’ clinical placement experiences provide important knowledge and insight for educators in terms of education and support for future student nurses going into situations with short clinical placement hours. The conclusion highlights the implications of the findings and recommendations and the need for further support and education for newly qualified nurses after the pandemic.

**Study registration details:**

The study was approved by the Norwegian Social Data Service (project number 396247). The registration date was 2021–11–04.

**Tweetable abstract:**

Transition-related challenges became more prominent during the coronavirus disease 2019 pandemic than during non-pandemic times.

## What is already known

• Many newly qualified nurses experience transition challenges because they feel unprepared for the reality of the nursing profession owing to the theory-practice gap.
• Many newly qualified nurses are leaving the profession due to transition-related challenges.
• Facilitating the transition from student nurses to newly qualified nurses is important to ensure safe and effective patient care.

## What this paper adds

• The restrictions on clinical placement education due to the coronavirus disease 2019 pandemic have been challenging for student nurses transitioning to newly qualified nurses.
• Newly qualified nurses could be offered a transition programme.

## 1 Background

Many newly qualified nurses are experiencing student-to-newly-qualified nurse transition-related challenges (Kox et al., 2020; Masso et al., 2022; Reebals et al., 2022; Rush et al., 2019; Visiers-Jiménez et al., 2022; Palese et al., 2022). Transition-related challenges occur due to a mismatch of education and clinical placements, indicating that newly qualified nurses feel unprepared for the reality of the profession due to the theory-practice gap (Kox et al., 2020; Masso et al., 2022; Reebals et al., 2022; Rush et al., 2019). For example, in the reviews by AlMekkawi and El Khalil (2020), Masso et al. (2022), Rush et al. (2019), and a comparative cross-sectional study by Liou et al. (2020), newly qualified nurses reported perceived inadequate knowledge and clinical skills in dealing with critical situations. Furthermore, in a comparative and cross-sectional study of six European countries by Palese et al. (2022), newly qualified nurses scored the lowest points on proficiency in discussing openly and solving problems. Transition challenges amongst newly qualified nurses might have profound consequences for the profession and patient care (Sultana, 2017). Despite efforts to reduce this theory-practice gap by incorporating more clinical placement hours into nursing education (Directive, 2013), many newly qualified nurses still experience unpreparedness after graduation (Masso et al., 2022). Consequently, many newly qualified nurses have the intention to leave the profession either temporarily or permanently (Visiers-Jiménez et al., 2022; Kovner et al., 2014). In addition, nurse shortages are a global challenge and contribute to increased workload and burnout for practising nurses and an eventual reduction in the provision of patient care (WHO, 2020). Therefore, nurse retention is necessary to ensure appropriate access to health care and patient safety (WHO, 2020; Buchan et al., 2015).

In the spring of 2020, student nurses abruptly shifted to remote education in response to the global coronavirus disease 2019 pandemic and missed clinical placement hours and experiences (Palese et al., 2022; Smith et al., 2021; Chinwendu et al., 2021). Consequently, the transition from student to newly qualified nurse became arduous, as it was difficult for them to adjust to their new role (Palese et al., 2022; Smith et al., 2021; Bani et al., 2022; Fernández-Basanta et al., 2022; Matlhaba and Khunou, 2022). However, transition-related challenges due to disrupted clinical placement education caused by the coronavirus disease 2019 pandemic during nursing education remain insufficiently explored (Palese et al., 2022; Smith et al., 2021; Bani et al., 2022; Matlhaba and Khunou, 2022; Luo et al., 2023; El Hussein et al., 2023). Therefore, this qualitative study explores the transition-related challenges due to disrupted clinical placement education in a time of crisis.

The new practise following the World Health Organization (2020) social distancing recommendations resulted in many cancelled clinical placement hours, and student nurses missed opportunities for clinical placement education (Palese et al., 2022; Dziurka et al., 2022; Espin et al., 2021). Instead of providing direct patient care and attending to diverse clinical placement situations, many nursing programmes found alternative solutions, such as digital- and simulation-based education (Palese et al., 2022; Chinwendu et al., 2021; El Hussein et al., 2023; Egilsdottir et al., 2022) to ensure that students received alternative clinical placement hours to be able to graduate and become registered nurses (Dziurka et al., 2022; Fogg et al., 2020; Thirsk et al., 2022; Utli and Yurt, 2022). In the Norwegian context, as in other countries (Palese et al., 2022; Smith et al., 2021; Thirsk et al., 2022; Liesveld et al., 2023), national guidelines (KUD, 2020) recommend that students should not be penalised or have their progress in their clinical placement education delayed due to restrictions or limitations on clinical placement hours. However, the disruptions in traditional clinical placement hours caused by the coronavirus disease 2019 pandemic resulted in many student nurses with less experience and hands-on training opportunities than previous generations of newly qualified nurses (Palese et al., 2022; Luo et al., 2023; El Hussein et al., 2023; Godbold et al., 2021).

A detailed and nuanced understanding of the complexity of transition-related challenges during the coronavirus disease 2019 pandemic is needed to retain newly qualified nurses during crises (Smith et al., 2021; Bani et al., 2022; Fernández-Basanta et al., 2022). Further, recent literature emphasises the need for research on transition-related challenges during the coronavirus disease 2019 pandemic to plan for quick practicable support in the event of new pandemics or crises and to find ways to support newly qualified nurses as they navigate their new role (Bani et al., 2022; Matlhaba and Khunou, 2022; Luo et al., 2023). These efforts would have long-term benefits for newly qualified nurses and the healthcare system as a whole (Bani et al., 2022). To help address the knowledge gap, we explored transition-related challenges that arise while facilitating the transition from student to newly qualified nurse when educational clinical placements are disrupted. Precisely, we explored the challenges faced by student nurses because of the lack of clinical placements due to the coronavirus disease 2019 pandemic. This study also included a self-assessment of the participants’ ability to provide appropriate, quality care as newly qualified nurses.

### 1.1 Conceptual framework

This study is underpinned by Benner (1984) ‘From Novice to Expert’ theory of competence development, which posits that nurses progress through five stages of development from novice to expert. The five stages of Benner (1984) theory provide a framework for understanding the progression of nurses' clinical knowledge and competence. Novices, who are new to the field with little or no clinical experience, rely heavily on rules and guidelines to guide their actions. In this stage, student nurses become familiar with new clinical placement settings. Advanced beginners are those who have acquired some experience and are beginning to understand the underlying principles of their work. In general, this stage begins with a newly qualified nurse. Competent nurses understand the principles well and can apply them in various situations, making appropriate decisions and taking appropriate action without relying on rules or guidelines. Proficient nurses have a high level of skill and knowledge and can anticipate and adapt to changing situations, while expert nurses have a deep understanding of the underlying principles of their work and can apply them to various situations.

### 1.2 Aim of the study

This study aimed to explore the experiences of newly qualified nurses regarding transition-related challenges from student-to-newly-qualified nurse status during the coronavirus disease 2019 pandemic. The following research question was addressed: how did the coronavirus disease 2019 pandemic affect the clinical placement of student nurses and their workplace experiences as newly qualified nurses?

## 2 Methods

### 2.1 Design

This study is the first part of a larger investigation exploring student nurses’ clinical placement education during the coronavirus disease 2019 pandemic as well as the experiences of newly qualified nurses in their workplace after completing their education during the coronavirus disease 2019 pandemic. The current study employed a qualitative exploratory and descriptive research design (Polit and Beck, 2020) to understand the challenges associated with the transition to the clinical placement setting as a training, learning, and workplace environment during a pandemic. Additionally, this study adopted an interpretive and constructive paradigm that takes into account the thoughts and experiences of the participants while considering the researchers’ reflexive influence on the interpretations of data (Braun and Clarke, 2006, Braun and Clarke, 2019).

### 2.2 Study participants and settings

This study recruited newly qualified nurses who had graduated from a university in southern Norway in June 2021 with a nursing bachelor's programme covering 180 European Credit and Transfer and Accumulation System European credits; these nurses had completed a major part of their education during the coronavirus disease 2019 pandemic. We used purposive sampling to enhance information power (Malterud et al., 2015), and we invited potential participants (*n* = 88) by email, explaining the study's purpose. The final data analysis included 10 newly qualified nurses who had been working directly in patient care for 9 months after graduation. We defined newly qualified nurses as having <3 years of post-graduation experience (Masso et al., 2022).

During their first year of education, all the participants had 180 h of clinical placement at a nursing home or home care setting. At the onset of the coronavirus disease 2019 pandemic in March 2020, all the participants were in their second year of education, and the focus of their clinical placement education was acute, critical, and chronically ill hospitalised patients. The nursing educational programme responded to the coronavirus disease 2019 pandemic following the guidelines of the World Health Organization and the national-specific guidelines regarding the pandemic. Clinical hospital education was suspended or restricted to safeguard physical distance and to protect the students and faculty, as well as the patients and staff, from the pandemic. Due to the pandemic, clinical placement education was disrupted during the second year of education. In their third and last year of education, most of the students participated in physical clinical placements (Table 1).

Owing to the restrictions in placement education, the students were encouraged by the educational institution to choose one of the following alternatives to clinical placement hours during their second and third years of education (KUD, 2020): (1) losing clinical placement hours entirely or (2) working as health care assistants. Working as health care assistants was an alternative for students who already had a salaried job as an assistant within the healthcare sector during their nursing education. Additionally, some participants had faced a sporadic loss of clinical placement hours due to coronavirus disease 2019 testing and waiting for test results (Table 1). Online learning became a substitute for clinical placement education. Since graduating, the participants have worked in various clinical placements, hospital wards, and municipal institutions as newly qualified nurses (Table 1).

**Table 1:** Participants’ demographic profile.

Participants	Clinical placement hours during second year of education	Health care assistant role as a substitute for ordinary clinical placement hours during second year of education	Clinical placement hours during third year of education	Health care assistant role as a substitute for ordinary clinical placement hours during third year of education	Working as a newly qualified nurse
**1**	Medical hospital ward: missed 25% Surgical hospital ward: missed 100%	Medical hospital ward: none substitution of the 25% Surgical hospital ward: 16,67% substitution in a medical placement ward	Nursing home setting: missed 50%	Nursing home setting: none substitution of the 50%	Elderly care
**2**	Medical hospital ward: missed 100%	Medical hospital ward: 25% substitution in a community service centre	Nursing home setting and psychiatric setting: no missed hours	Not relevant	Elderly care
**3**	Medical hospital ward: missed 100%	Medical hospital ward: 50% substitution in a medical hospital ward	Nursing home setting and psychiatric setting: no missed hours	Not relevant	Acute, critical, chronically ill patients
**4**	Medical hospital ward: missed 100%	Medical hospital ward: 50% substitution in a psychiatric hospital ward	Nursing home setting and psychiatric setting: no missed hours	Not relevant	Acute, critical, chronically ill patients
**5**	Medical hospital ward: missed 25%	Medical hospital ward: no substitution of the 25%	Nursing home setting and psychiatric setting: no missed hours	Not relevant	Acute, critical, chronically ill patients
**6**	Medical and surgical hospital wards: missed 3.7%	Medical and hospital wards: no substitution of the 3.7%	Nursing home setting and psychiatric setting: missed 3.7%	Nursing home setting and psychiatric setting: no substitution of the 3.7%	Acute, critical, chronically ill patients
**7**	Health promotion observation practice: missed 100%	Health promotion observation practice: no substitution of the 100%	Nursing home setting and psychiatric setting: no missed hours	Not relevant	Elderly care
**8**	Surgical hospital ward: missed 100%	Surgical hospital ward: 50% substitution in an intensive hospital ward	Nursing home setting: missed 50%	Nursing home settings: no substitution of the 50%	Mental health
**9**	Medical and surgical hospital ward: missed 1.4%	Medical and surgical hospital ward: no substitution of the 1.4%	Nursing home and psychiatric setting: missed 1.4%	Nursing home and psychiatric setting: none substitution of the 1.4%	Elderly care
**10**	Surgical hospital ward: missed 25% Medical hospital ward: missed 100%	Surgical hospital ward: no substitution of the 25% Medical hospital ward: 50% substitution in a medical day ward	Nursing home setting and psychiatric setting: no missed hours	Not relevant	Elderly care

### 2.3 Data collection

Data collection occurred from April to May 2022 through individual interviews. We chose interviews as the data collection method to gain a comprehensive understanding of the phenomenon of interest (Polit and Beck, 2020). The first author, who has a background in nursing education and qualitative research, conducted the comprehensive interviews using a semi-structured interview guide developed by the research team. The interview guide aimed to explore various aspects related to the pandemic (Table 2). The participants were encouraged to speak freely about their experiences, and follow-up questions were asked when appropriate. Due to pandemic restrictions, all interviews took place online via electronic resources (Zoom) at a mutually agreed-upon time. All the participants and the researcher used both video and audio, and the interview format facilitated two-way communication, enabling a comprehensive discussion of relevant topics (Polit and Beck, 2020). Each interview lasted approximately 90–120 min; the interviews were audio-recorded and transcribed verbatim.

**Table 2:** Focus of the interviews with examples of questions.

Focus of the individual interviews concerning the clinical placement education as a student nurse:	Focus of the individual interviews concerning the transition into professional workplace as a newly qualified nurse:
The interview guide aimed to explore various aspects related to training, learning opportunities, and experiences related to the coronavirus disease 2019 pandemic.Examples of interview questions: • How did you experience being a student nurse with limited opportunities for physical presence during clinical placement? • How was the follow-up provided by registered nurse mentors during clinical placement that occurred during the pandemic? • How did you experience possibilities for learning to act as a health care assistant? • How did you experience connecting theory to practice when physical presence was limited?	The interview guide aimed to explore perceptions and experiences related to the transition to professional work during the coronavirus disease 2019 pandemic as newly qualified nurses.Examples of interview questions: • How did you perceive your nursing competence as a newly qualified nurse being educated during a pandemic, considering clinical placements being to substituted, to some extent, with online learning and health care assistant jobs? • Did you experience any learning outcomes as particularly challenging? Please describe. • How did you experience performing practical nursing skills on patients after completing your education?

### 2.4 Ethical considerations

The study was performed in accordance with the guidelines of the National Committee for Research Ethics in the Social Sciences and the Humanities (NESH, 2016) and approved by the Norwegian Social Data Service (project number 396247) and the university (which provided access to the participants’ email addresses stored in the university's data system). All participants were informed in writing about the study and that their participation was voluntary. Informed consent was obtained when the participants answered the email about their participation. Confidentiality, anonymity, and the right to withdraw participation at any time were guaranteed. To ensure confidentiality, participant characteristics such as age; sex; and the name of the educational institution, institutions, or wards in which they were working were obscured. The researcher who conducted the interviews had not taught or supervised the participants previously. Recordings, consent forms, and transcribed texts were stored on the university's research server.

### 2.5 Data analysis

For data analysis, the inductive reflexive thematic analysis method, which is a data-driven approach without any pre-existing theoretical understanding or coding frame, developed by Braun and Clarke, 2006, Braun and Clarke, 2019 was used. The analysis was reflexive, recursive, and iterative, allowing for flexibility in moving back and forth through the different phases of the analysis process as necessary (Braun and Clarke, 2006, Braun and Clarke, 2019). No software was used to manage the data.

The analysis was carried out in the following steps: (1) familiarisation with the data by re-reading the transcribed text several times and identifying patterns of interest; (2) identifying meaning units relevant to the research question and generating initial codes; (3) grouping similar codes to generate preliminary themes and sub-themes; (4) ensuring that the preliminary themes and sub-themes contained consistently coded meaning units; (5) naming each defined theme and providing a brief description that captured its essence, and (6) describing the defined themes and sub-themes as part of the final manuscript. The first author extracted the data, and the last author validated the data; any disagreements were resolved by discussion until a mutual agreement was reached. A pre-analysis was conducted involving the research team members to discuss the meaning units, codes, sub-themes, and themes until a consensus was reached. Finally, the research group discussed the findings of the study and how they correlated with the themes, categories, codes, and meaning units arising from the data analysis. Quotations were identified from the data and adjusted to provide a clear understanding of what was said.

### 2.6 Rigour

To ensure trustworthiness, the study followed four criteria (Lincoln and Guba, 1985): credibility, dependability, transferability, and confirmability. All interviews were conducted by a researcher (MR) who had sufficient training and experience in conducting semi-structured interviews using Zoom. An interview guide was used to ensure consistency in the data collection. Dependability was ensured by providing detailed descriptions of the data collection and analysis processes. To enhance transferability, detailed descriptions of the research process were provided. The validity of the findings was ensured by employing triangulation and discussing the analysis of the data with the research team. The participants were purposefully selected to provide the highest amount of information, and information power was reached owing to the sample specificity and quality of the dialogue (Malterud et al., 2015). The study gathered unique knowledge and insights into the experiences of 10 newly qualified nurses with different ranges of clinical education placements during the pandemic. Relevant quotes were selected and effectively integrated into the manuscript to exemplify the content.

## 3 Findings

The study revealed three main themes and six sub-themes that described the challenges faced by newly qualified nurses during their transition period due to the lack of clinical placement experiences during their education in the coronavirus disease 2019 pandemic (Table 3).

**Table 3:** Themes and sub-themes.

Themes	Sub-themes
Relational aspects of nursing became hidden	Patient contact
	Contact with next of kin
Practical aspects of nursing became hidden	Practical nursing skills
	Medication calculation skills
The pedagogical plan of teaching and learning was inadequate	Provision of supervision
	Complementary between theory and practice

### 3.1 Relational aspects of nursing became hidden

The first theme, ‘relational aspects of nursing became hidden’, highlighted how the participants missed out on opportunities to interact with patients and their next of kin during their clinical placement. This affected their ability to establish relationships with patients and their families as newly qualified nurses.

#### 3.1.1 Patient contact

The sub-theme of patient contact described how social distancing measures and full protective clothing worn before patient contact prevented the participants from engaging with patients. The lack of elective patients admitted to the hospital also affected their learning. One participant who attended most of the clinical placement hours in hospital wards stated the following:

*‘It was not the normal patient base in the hospital wards during my clinical placements.’ (5)*

The lack of opportunities for patient contact during clinical placement education influenced their ability to establish patient contact as expected by a newly qualified nurse. Some participants experienced stress in meeting and following up with patients due to a lack of experience in educational placements. One participant who had never had any experience at a medical hospital ward during education expressed her situation in the following way:

*‘I would have had experience with several patient cases that could have been useful in my current job as a registered nurse if my clinical placement education at the hospital had been completed. Meeting patients via placements is very important in order to be able to meet different patient situations as a fully qualified nurse.’ (2)*

#### 3.1.2 Contact with the next of kin

The sub-theme of contact with next of kin revealed how the participants lacked experience interacting with patients’ families during their clinical placement education, causing uncertainty in their new role as newly qualified nurses:

*‘As a newly qualified nurse, I experienced uncertainty in meeting with the patients’ next of kin, both in dealing with them directly and also when trying to care for them. This was a result of having had little contact with the next of kin as a student during my clinical placement education. It is tough and demanding to be in situations with the next of kin as a nurse, particularly because I have had limited previous experience as a student.’ (5)*

### 3.2 Practical aspects of nursing became hidden

The second theme, ‘practical aspects of nursing became hidden’, described how practical nursing skills and medication calculation skills were inadequately taught, learned, and practised during clinical placement education, further affecting the participants’ practise as newly qualified nurses.

#### 3.2.1 Practical nursing skills

The sub-theme of practical nursing skills explained how reduced physical presence and restrictions on health care assistants performing practical skills affected the participants’ opportunities to learn practical skills. Participants in the health care assistant role were not allowed to perform practical skills. Consequently, the lack of training opportunities adversely affected the participants’ confidence in their practical skill performance. A participant who worked as a health care assistant in a medical hospital ward expressed the following:

*‘In medical practice I was mostly left on my own and was not taught or shown the nursing tasks in the role of a health care assistant.’ (10)*

The participants found peripheral vein cannulation particularly challenging to learn due to the lack of training opportunities. Insufficient competence in practical nursing skills affected the participants’ self-satisfaction with performing the skill. One participant who missed all clinical placement hours in a medical hospital ward said the following:

*‘It was experienced as a failure to never master vein cannulation on a patient.’ (4)*

Lack of competence in practical nursing skills made it challenging for the participants, as newly qualified nurses, to perform such skills on patients. As newly qualified nurses, the participants experienced a connection between mastery of practical nursing skills and the training they received as students during clinical placement education. This connection was stated by the only participant who had fulfilled all the clinical placement hours as a student in the hospital wards:

*‘Practical skills learned during education, which are repeated in the department, are mastered’. (7)*

Regarding the practical skill of peripheral vein cannulation, the newly qualified nurses specifically experienced a lack of competence. One participant who worked as a health care assistant during placement education in a medical ward stated the following:

*‘As a newly qualified nurse, I am unsure about inserting a peripheral vein cannula, and especially so in acute situations’. (3)*

Lack of competence in peripheral vein cannulation made it challenging for the participants, as newly qualified nurses, to perform such skills on patients, giving them an unpleasant experience. One participant acknowledged the following:

*‘Lack of mastering insertion a peripheral vein cannulation stays with you for a long time and stands out as a particularly unpleasant nursing experience’. (2)*

Furthermore, it was discovered that a lack of mastery of peripheral vein cannulation as a newly qualified nurse had negative consequences for further learning of the skill, where the practical skill was left to others to perform:

*‘Lack of confidence in peripheral vein cannulation cases lead to asking others perform the task’. (2)*

#### 3.2.2 Medication calculation skills

This sub-theme explained how the participants received limited training in medication calculation skills due to missed clinical placement hours. Furthermore, most medication calculations were simple and not challenging; the participants used some medications in the ward that were pre-mixed from pharmacies, and the nurses used special tools to assist them with medication calculations. The participant who fulfilled all the clinical placement hours in hospital wards as a student expressed the following:

*‘In the clinical placement at the hospital, an infusion pump was used, so there was no need to make medication calculations; it was enough to simply set the pump to work’. (7)*

In addition, the lack of supervision hindered the participants’ learning of medication calculation skills during the clinical placement, as stated below:

*‘Supervising nurses gave little attention to what would be the correct drop rate’. (7)*

The participants who undertook the role of health care assistant during their clinical placement did not get the opportunity to perform medication calculations related to real patient situations. One piece of information said the following:

*‘I did not have the opportunity to access the medicine room in my role as a health care assistant on the ward.’ (1)*

The participants as newly qualified nurses found medication calculations challenging to master and highlighted that the topic should have been given more attention during education. Furthermore, the participants spent more time than necessary when advanced medication calculations were needed, as stated below:

*‘Even simple tasks in medication calculations can be difficult to perform, and I do not have knowledge in advanced medication calculations.’ (8)*

It was also pointed out that knowledge of medication calculations was continuously required of newly qualified nurses in clinical placements and that repetitive training in medication calculations was a prerequisite for maintaining safe and correct handling. One participant expressed the following:

*‘It is useful to continue training on medication calculations and to solve practice cases even as a newly qualified nurse.’ (10)*

The participants experienced the challenge of supervising students in their clinical placement when performing medication calculations as they lacked the knowledge:

*‘It is difficult to teach students medication calculations when you cannot do it yourself.’ (2)*

### 3.3 Pedagogical plan for teaching and learning was inadequate

The third theme, ‘the pedagogical plan of teaching and learning was inadequate’, revealed how the provision of supervision and the complementary relationship between theory and practise were inadequate during clinical placement education. The participants lacked the necessary support and guidance to learn effectively, and this affected their learning outcomes as newly qualified nurses.

#### 3.3.1 Provision of supervision

The sub-theme of provision of supervision described how the participants lacked supervision from experienced registered nurses and how the supervision they received was inadequate. Missing or reduced time spent in clinical placement education denoted that the participants lost supervision time with nurse mentors. However, completion of clinical placement education did not mean that the participants were consistently supervised by their nurse mentors. One participant stated the following:

*‘I didn't have a nurse mentor to go with as the nurse was ill for almost the entire clinical placement period; it was disturbing.’ (2)*

There was often no plan for who would supervise the participants in the absence of a given mentor during the clinical placement, and the participants had to be mentored by different nurses. The participants were required to convince the nurses about their abilities and knowledge. The department considered the participants who worked as health care assistants during their clinical placement education as employees, and their learning and competence development were not areas of focus. One participant stated the following:

*‘I received no supervision from a nurse when I was working as a health care assistant.’ (3)*

The participants experienced the need for formal development as newly qualified nurses, and very few participants had the opportunity for this. One participant said the following:

*‘I have tried to take the initiative to enhance my skills, but I was not heard.’ (2)*

Generally, the participants had positive experiences asking colleagues for help to acquire knowledge; however, some experienced colleagues questioned their competence level after graduation. Two participants stated the following:

*‘As a newly qualified nurse, I have asked for help when performing practical skills, which has been well received by colleagues.’ (1)*

*‘There is an expectation from registered nurses that we shall know everything when we have graduated as nurses.’ (9)*

#### 3.3.2 Complementarity between theory and practise

The sub-theme of complementarity between theory and practise showed how the participants felt a mismatch between their theoretical knowledge and the practical skills they were expected to perform as newly qualified nurses. The participants found that the lack of physical presence during clinical placement education inhibited their learning processes. Without experience in the clinical placement context, it was challenging for the participants as students to relate theoretical knowledge to practice. Furthermore, the participants stated that the knowledge acquired through theoretical subjects in school was quickly forgotten and could not be related to practice. Additionally, the participants who were mostly present during their clinical placement education experienced challenges in connecting theoretical and practical knowledge, given the limited access to patients and learning situations owing to the prevalence of infection control practises during the pandemic. Two participants who only missed some of the clinical placement hours in the hospital wards stated the following:

*‘It was a bit limited what we were allowed to participate in during clinical placement hours due to the pandemic; we had to be careful not to spread infection’. (9)*

*‘It is challenging to link theory to practice when you are not allowed to be in practice, and it is difficult to gain an understanding of reality when you are not there’. (6)*

As health care assistants, the participants experienced learning outcomes that were not within the scope of the role of student nurse. The role of health care assistant was considered a pastime by the participants:

*‘The role as a health care assistant was intended to fill up the clinical placement hours’ (3)*

The participants felt overwhelmed and unsure when starting their work because they lacked the knowledge and experience necessary to act as newly qualified nurses. The lack of clinical placement experience in surgical and medical hospital wards was responsible for some participants not choosing a hospital ward context as their place of work. One participant who had lost all clinical placement hours in a surgical ward stated the following:

*‘I think the transition from being a student nurse to a newly qualified nurse in a hospital ward would be vast if clinical placement education in hospital wards (medicine and surgery) were not completed.’ (8)*

Despite it being demanding for the participants to start working as newly qualified nurses in a nursing field they lack experience in, some of them chose to work in a nursing field where they previously, as student nurses, had no experience after completing their education. One participant stated the following:

*‘I am quite fearless, and I like to take things on the spur of the moment and also to be in stressful situations. Therefore, I am not afraid of starting to work in a hospital ward even if a placement education was missed.’ (6)*

However, some participants changed their place of work as newly qualified nurses because it was too demanding to handle and master the tasks, practical skills, and knowledge they lacked as student nurses. For the participants, it was challenging to apply knowledge in clinical placement situations because they lacked experience and had not previously encountered similar situations. One participant who attended most of the clinical placement hours during education stated the following:

*‘I changed jobs when I realised that my skills were not good enough at my first chosen workplace.’ (5)*

## 4 Discussion

This study analysed the data obtained from interviews with newly qualified nurses working in different clinical placement settings who shared their experiences related to their roles as students and newly qualified nurses. This study contributes to the existing knowledge on transition-related challenges amongst newly qualified nurses by providing new insights on the transition from student to newly qualified nurses during the coronavirus disease 2019 pandemic. It answers the call of the current literature for research and knowledge on transition-related challenges to improve nursing preparedness during extreme conditions (Palese et al., 2022; Liesveld et al., 2023). Furthermore, this might contribute to improving the retention of newly qualified nurses, which is a vital part of the nursing profession (WHO, 2020; Matlhaba and Khunou, 2022).

As observed in previous research, the participants in our study had restricted opportunities for direct patient care and hands-on training during their education since reducing the spread of the coronavirus disease 2019 virus was prioritised (Smith et al., 2021; El Hussein et al., 2023; Dziurka et al., 2022; Espin et al., 2021; Ramos-Morcillo et al., 2020; Calica and Paterson, 2023). Additionally, to reduce the loss of clinical placement hours and to continue their course of education, these hours were substituted by regular work as health care assistants (KUD, 2020). The participants were not motivated by the solution of being health care assistants during clinical placement hours because they had to work as employees whose tasks and learning outcomes were not within their primary job scope. However, these findings contradicted the reports of the study by Casafont et al. (2021), who stated that student nurses found health care assistant roles to be valuable in terms of skill and knowledge acquisition. The participants in our study were in their second year of education when they had to work as health care assistants, whereas, in the study by Casafont et al. (2021), the participants were in the last part of their education and seemed more independent and prepared for the nursing role. In addition, Benner (1984), in ‘Novice to Expert’, justified the need for clinical placement experiences to ensure competent independent nursing practice; this point raises concerns about the disruption of students’ clinical placement experiences early in their education during the crisis and its effects on education progression and the transition to newly qualified nurses.

Overall, our findings revealed newly qualified nurses lacked readiness for practise due to disrupted clinical placement hours and experiences in light of the perceived knowledge deficits related to relational and practical aspects of nursing. For instance, student nurses educated during the coronavirus disease 2019 pandemic felt unprepared to become registered nurses due to inadequate clinical placement experiences (Liesveld et al., 2023; Palomino et al., 2023). This finding illustrates that clinical placement experience is an important component of nursing education; it allows the participants to train, learn, and experience the knowledge and skills needed to engage in professional and competent nursing (Benner, 1984; Ravik and Bjørk, 2023). Since the participants lacked knowledge of the key aspects of nursing due to disrupted clinical placement experiences, they did not have the knowledge that Benner (1984) described as necessary to build on as newly qualified nurses. Sufficient clinical placement training and learning are prerequisites for developing competent nursing practice (Palese et al., 2022; Benner, 1984; Ravik and Bjørk, 2023). It was, however, important to continue educating student nurses during the pandemic to ensure that newly qualified nurses met the growing demand for healthcare services (Palese et al., 2022; WHO, 2020).

The participants considered the relational aspects of nursing important for patient safety when they described the challenges associated with interacting with patients and their families as newly qualified nurses. They underscored the importance of interacting with patients and their families in educational clinical placements to understand patients’ needs and concerns and to not overlook important information about the patient's health as newly qualified nurses. These findings correspond with the findings of Woo and Newman (2020), namely that experiencing relationships with patients and their families was important for students during clinical placements to learn about patients’ needs and preferences and how to provide appropriate and compassionate care as newly qualified nurses. Palomino et al. (2023) reported that it was challenging to learn and develop communication skills owing to the absence of face-to-face contact with patients during clinical placement education. Interestingly, Masso et al. (2022), in their review of 45 studies (905 original studies from 1989 to 2019) on transition challenges amongst newly qualified nurses, did not clearly identify the challenges corresponding to relational aspects with patients and their families as a crucial transition-related challenge in non-pandemic times. These data highlight the vital need for relational aspects of nursing to be strengthened during any pandemic to equip newly qualified nurses with the appropriate skills (Bani et al., 2022).

The participants experienced challenges related to their educational and clinical placement experiences that were restricted by the coronavirus disease 2019 pandemic. In turn, they needed more time to adjust to their roles as newly qualified nurses when performing practical aspects (e.g. practical nursing skills and medication calculation skills). Although the participants attributed this challenge to the disruption of their clinical placement experiences during their education, the practical aspects of nursing have been reported to be transition-related challenges for decades (Masso et al., 2022; Luokkamäki et al., 2020). Several studies have reported that newly qualified nurses need to re-learn the practical aspects to effectively cope with the demands of the skills in clinical placements (Alandajani et al., 2022; Dover et al., 2019; Mlambo et al., 2021; Mulac et al., 2022; Yalçınlı et al., 2019). Notably, the difficulties in practical nursing skills might be attributed to the complexity and preciseness they demand from registered nurses, as indicated in the model of practical skill performance (Bjørk and Kirkevold, 2000). Along the same line, the complexities of medication calculation are related to difficulties in the practical aspect of nursing (El Hussein et al., 2023; Elonen et al., 2021). Although such aspects are a mainstay for registered nurses in clinical placements (Mulac et al., 2022; Currie et al., 2023), student nurses’ training and learning are not prioritised in academic settings in general (Hilleren et al., 2022; Stolic, 2014; Stolic et al., 2022). The participants in our study particularly spoke about the practical skill of peripheral vein cannulation as challenging to learn, consistent with findings reported in previous research (Bridge et al., 2022; Ravik et al., 2017; Ravik et al., 2015). This finding suggests that the practical aspects of nursing must be prioritised in clinical placement education, as they allow students to take on various clinical situations as newly qualified nurses (Ravik and Bjørk, 2023; Currie et al., 2023). Furthermore, dealing with actual medical disorders cannot be replaced by training and learning in academic settings, such as in a simulation setting (Ravik and Bjørk, 2023; Bridge et al., 2022; Ravik et al., 2015; Matchim and Kongsuwan, 2015). Therefore, Benner (1984) emphasises the importance of contextual factors in achieving proficiency above and beyond individual factors.

Altogether, the current study's findings indicated that a lack of confidence in the practical aspects of nursing was connected to knowledge deficits. Moreover, the participants reported that they felt overwhelmed by their role as newly qualified nurses due to a lack of confidence in the practical aspect of nursing. The lack of confidence and feeling overwhelmed are not novel challenges, as these have been mentioned in previous research. Newly qualified nurses felt like they were a burden to their colleagues because of their lack of confidence in their abilities and fear of making mistakes in pre- and intra-pandemic times (Matlhaba and Khunou, 2022; Mathebula et al., 2022). The gap between the student nursing role and the autonomous nursing role is a risk factor for newly qualified nurses who cannot cope with the practical aspects (Reebals et al., 2022). The knowledge deficits in the practical aspects of nursing can be attributed to this gap, which can lead to inadequate diagnostic and medical treatment and patient safety (Mulac et al., 2020; Piper et al., 2018; Zamanzadeh et al., 2015).

Our participants experienced a theory-practice gap. This means that it was challenging to fully understand what was learned in the academic setting (See et al., 2023) and to apply it in clinical placement settings due to inadequacies in the pedagogical plan of teaching and learning during the coronavirus disease 2019 pandemic. These findings were in line with the results of previous research on the importance of pedagogical plans, such as supervision during the education and transition periods, in developing confident and competent healthcare practitioners (Palese et al., 2022; Bani et al., 2022; El Hussein et al., 2023; Barisone et al., 2022; Laugaland et al., 2021). Benner (1984) emphasised that the most skilled nursing performance can be achieved in a supportive clinical placement setting. In the quantitative study by Palese et al. (2022), it was reported that last-year student nurses who received supervision by clinical nurses during placement education that occurred during the coronavirus disease 2019 pandemic, perceived themselves as being well prepared as newly qualified nurses. Furthermore, in a literature study by van Rooyen et al. (2018), the authors reported that newly qualified nurses had increased job satisfaction when they had opportunities to learn and advance their knowledge, understanding, and techniques with sufficient support. In our study, the newly qualified nurses had opportunities to provide safe and appropriate care because they were helped in identifying areas for improvement and further learning (van Rooyen et al., 2018). The literature demonstrates that many healthcare professionals were not provided appropriate support during the coronavirus disease 2019 pandemic due to the heavy workload amongst registered nurses and the nature of the pandemic (Joseph et al., 2022). Additionally, many registered nurses left the profession during the pandemic (Kleier et al., 2022). This might be the reason why the participants in our study experienced inadequate formal supervision during their clinical placement.

Further, the participants had to adapt to a rapidly changing clinical placement setting due to the pandemic crisis, thereby facing uncertainty. These experiences might help the participants build resilience and prepare for the challenges they will face as newly qualified nurses (Palese et al., 2022; Godbold et al., 2021). However, many participants (as newly qualified nurses) reported a theory-practice gap that was extensively managed; they did not choose a nursing field to work in as they did not acquire experience from their clinical placement during their education. This is consistent with the reports of Matlhaba and Khunou (2022) who found that limited clinical placement experiences during education contributed to a difference between idealistic expectations and the reality experienced amongst newly qualified nurses. Further, Liesveld et al. (2023) suggested that clinical placement experiences during education are predictors of the competence of newly qualified nurses. This finding indicates that the participants lost opportunities to become familiar with the registered nurse's role in the clinical placement setting (Fernández-Basanta et al., 2022; Espin et al., 2021). They could not experience actual situations through which a nurse can improve, elaborate on, or confirm their knowledge and understanding to improve their competence (Benner, 1984). Consequently, insecurity in providing patient care, burnout, dissatisfaction, and high turnover rates amongst newly qualified nurses could lead to a shortage of registered nurses and increase costs associated with recruiting and training newly qualified nurses (Kox et al., 2020; Bani et al., 2022; Aldosari et al., 2021).

### 4.1 Limitations

There are some limitations to our study that need to be acknowledged. First, it only included participants from one country, and the findings may not be transferable to other settings or cultures. Second, our sample size was small, and we cannot rule out the possibility of selection bias. Third, our study relied on self-reported data from the participants, which may be subject to recall or social desirability bias. Finally, our study did not include the long-term influences of the pandemic on the transition experiences of newly qualified nurses from the undergraduate bachelor's programme in nursing. This may limit the transferability of the findings to other educational nursing programmes. Future research should explore these issues to better understand the challenges faced by newly qualified nurses during times of crisis.

In addition, there has been little focus on using the health care assistant role as a substitute for placement education and how patients are affected by transition-related challenges facing newly qualified nurses; future studies should address this issue.

## 5 Conclusions

As presented in our data, the participants received their education and started their professional experience as newly qualified nurses during the coronavirus disease 2019 pandemic. A disruption of clinical placement education illuminated transition-related challenges due to the lack of clinical placement experience. The transition-related challenges perceived by the participants as newly qualified nurses were linked to the training and learning opportunities in their clinical placement education. Many participants worked as health care assistants during clinical placement, and this was considered inadequate for acquiring knowledge and experience.

Transition-related challenges corresponded to a theory-practice gap and were perceived as knowledge deficits in relational and practical aspects of nursing and inadequacies in the pedagogical plan of teaching and learning. Transition-related challenges were responsible for determining newly qualified nurses’ choices in their workplace. The transition-related challenges faced by the participants as newly qualified nurses in our study demonstrate the need for clinical placement education despite crises like the coronavirus disease 2019 pandemic. Moreover, it is not recommended that student nurses be prevented from completing clinical placements since the lack of these placements had a negative influence on training, learning, and transition, including the skills to handle health emergencies as newly qualified nurses. Furthermore, we suggest that student nurses and newly qualified nurses be provided supervision and further learning to ensure safe and high-quality patient care, which should be prioritised in healthcare institutions, as feeling inadequate due to knowledge deficits is a primary reason why many student nurses and newly qualified nurses resign from their nursing roles (Reebals et al., 2022; Bani et al., 2022; Soerensen et al., 2023).

## CRediT authorship contribution statement

All authors agreed on the study design and worked together to design the interview guide. The first author collected data and was responsible for drafting the manuscript. The authors worked together on the analysis in the early and final phases of the analysis; the first and last authors significantly contributed to the data analysis. All authors critically reviewed the manuscript.

## Data availability statement

To access the dataset used and analysed during the current study, please contact the corresponding author.

## Funding sources

No external funding was received. This work was supported by the University of South-Eastern Norway. The funder had no role in the design of the study; collection, analysis, and interpretation of data; or the writing and publication of the manuscript.

## Declaration of Competing Interest

The authors declare that they have no known competing financial interests or personal relationships that could have appeared to influence the work reported in this paper.

## References

[bib0001] Alandajani A., Khalid B., Ng Y.G., Banakhar M. (2022). Knowledge and attitudes regarding medication errors among nurses: a cross-sectional study in major Jeddah hospitals. Nurs. Rep..

[bib0002] Aldosari N., Pryjmachuk S., Cooke H. (2021). Newly qualified nurses’ transition from learning to doing: a scoping review. Int. J. Nurs. Stud..

[bib0003] AlMekkawi M., El Khalil R. (2020). New graduate nurses’ readiness to practise: a narrative literature review. Health Prof. Educ..

[bib0004] Bani M. (2022). Jumping into the COVID-19 arena’: the professional transition into clinical practice of new graduate nurses in Italy at time of COVID-19. J. Clin. Nurs..

[bib0005] Barisone M. (2022). Nursing students’ clinical placement experiences during the Covid-19 pandemic: a phenomenological study. Nurs. Educ. Pract..

[bib0006] Benner P. (1984). From novice to expert: excellence and power in clinical nursing practice. XXVII.

[bib0007] Bjørk I.T., Kirkevold M. (2000). From simplicity to complexity: developing a model of practical skill performance in nursing. J. Clin. Nurs..

[bib0008] Braun V., Clarke V. (2006). Using thematic analysis in psychology. Qual. Res. Psychol..

[bib0009] Braun V., Clarke V. (2019). Reflecting on reflexive thematic analysis. Qual. Res. Sport Exerc. Health.

[bib0010] Bridge P. (2022). Simulated placements as partial replacement of clinical training time: a Delphi consensus study. Clin. Simul. Nurs..

[bib0011] Buchan J., Duffield C., Jordan A. (2015). Solving’ nursing shortages: do we need a New Agenda?. J. Nurs. Manag..

[bib0012] Calica K.A.N., Paterson R.E. (2023). The listening project: a qualitative study on the experiences of pre-registered nurses during the Covid-19 pandemic in Scotland. Heliyon.

[bib0013] Casafont C. (2021). Experiences of nursing students as healthcare aid during the COVID-19 pandemic in Spain: a phemonenological research study. Nurs. Educ. Today.

[bib0014] Chinwendu F.A., Stewart J., McFarlane-Stewart N., Rae T. (2021). COVID-19 pandemic effects on nursing education: looking through the lens of a developing country. Int. Nurs. Rev..

[bib0015] Currie J. (2023). A scoping review of clinical skill development of preregistration registered nurses in Australia and five other English-speaking countries. J. Clin. Nurs..

[bib0016] Directive. Directive 2013/55/EU of the European parliament and of the council of 20 November 2013 amending Directive 2005/36/EC on the recognition of professional qualifications and Regulations (EU) No 1024/2012 on administrative cooperation through the Internal Market Information System. Directive 2013/55/EU of the European parliament and of the council of 20 November 2013 amending Directive 2005/36/EC on the recognition of professional qualifications and Regulation (EU) No 1024/2012 on administrative cooperation through the internal market information system (the IMI Regulation)text with EEA relevance (europa.eu) (2013).

[bib0017] Dover N. (2019). A rapid review of educational preparedness of advanced clinical practitioners. J. Adv. Nurs..

[bib0018] Dziurka M. (2022). Clinical training during the COVID-19 pandemic: experiences of nursing students and implications for education. IJERPH.

[bib0019] Egilsdottir H.Ö. (2022). The value of a redesigned clinical course during COVID-19 pandemic: an explorative convergent mixed-methods study. BMC Nurs..

[bib0020] El Hussein M.T., Dosani A., Al-Wadeiah N. (2023). Final-year nursing students’ experiences during the COVID-19 pandemic: a scoping review. J. Nurs. Educ..

[bib0021] Elonen I. (2021). Medication calculation skills of graduating nursing students within European context. J. Clin. Nurs..

[bib0022] Espin S. (2021). Exploring the intersection between academic and professional practice during the COVID-19 pandemic: undergraduate and graduate nursing students’ experiences. Can. J. Nurs. Res..

[bib0023] Fernández-Basanta S., Espremáns-Cidón C., Movilla-Fernández M.J. (2022). Novice nurses’ transition to the clinical setting in the COVID-19 pandemic: a phenomenological hermeneutic study. Collegian.

[bib0024] Fogg N. (2020). Transitioning from direct care to virtual clinical experiences during the COVID-19 pandemic. J. Prof. Nurs..

[bib0025] Godbold R., Whiting L., Adams C., Naidu Y., Pattison N. (2021). The experiences of student nurses in a pandemic: a qualitative study. Nurs. Educ. Pract..

[bib0026] Hilleren I.H.S., Christiansen B., Bjørk I.T. (2022). Learning practical nursing skills in simulation centers – a narrative review. Int. J. Nurs. Stud. Adv..

[bib0027] Joseph H.B. (2022). Transitional challenges and role of preceptor among new nursing graduates. J. Caring Sci..

[bib0028] Kleier J.A. (2022). Professional commitment, resilience and intent to leave the profession among nurses during the COVID-19 pandemic - a descriptive study. J. Nurs. Manag..

[bib0029] Kovner C.T., Brewer C.S., Fatehi F., Jun J (2014). What does nurse turnover rate mean and what is the rate?. Policy Polit. Nurs. Pract..

[bib0030] Kox J.H.A.M. (2020). Reasons why Dutch novice nurses leave nursing: a qualitative approach. Nurs. Educ. Pract..

[bib0031] KUD. *Forskrift om endring i imidlertid forskrift om gjennomføring av utdanninger regulert av rammeplan mv. i forbindelse med utbruddet av Covid 19.* (2020).

[bib0032] Laugaland K.A. (2021). Nursing students’ experience with clinical placement in nursing homes: a focus group study. BMC Nurs..

[bib0033] Liesveld J., Rohr J., Petrovic K., Grohman S., Bourgeois C.L. (2023). Nursing student challenges during the COVID-19 pandemic from 2020 to 2021: a thematic analysis. Teach. Learn. Nurs..

[bib0034] Lincoln Y.S., Guba E.G. (1985).

[bib0035] Liou S., Liu H., Tsai S., Chu T., Cheng C. (2020). Performance competence of pregraduate nursing students and hospital nurses: a comparison study. J. Clin. Nurs..

[bib0036] Luo J., Luo L., Yang A., Cui M., Ma H. (2023). Clinical experiences of final-year nursing students during the COVID-19 pandemic: a systematic review and meta-synthesis. Nurse Educ. Today.

[bib0037] Luokkamäki S., Härkänen M., Saano S., Vehviläinen-Julkunen K. (2020). Registered nurses’ medication administration skills: a systematic review. Scand. J. Caring Sci..

[bib0038] Malterud K., Siersma V.D., Guassora A.D. (2015). Sample size in qualitative interview studies: guided by information power. Qual. Health Res..

[bib0039] Masso M., Sim J., Halcomb E., Thompson C. (2022). Practice readiness of new graduate nurses and factors influencing practice readiness: a scoping review of reviews. Int. J. Nurs. Stud..

[bib0040] Matchim Y., Kongsuwan W. (2015). Thai nursing students’ experiences when attending real life situations involving cardiac life support: a Phenomenological study. Nurs. Educ. Today.

[bib0041] Mathebula L., Downing C., Kearns I.J. (2022). Experiences of newly qualified professional nurses practising caring to patients at an academic hospital. Int. J. Africa Nurs. Sci..

[bib0042] Matlhaba K.L., Khunou S.H. (2022). Transition of graduate nurses from student to practice during the COVID-19 pandemic: integrative review. Int. J. Africa Nurs. Sci..

[bib0043] Mlambo M., Silén C., McGrath C. (2021). Lifelong learning and nurses’ continuing professional development, a metasynthesis of the literature. BMC Nurs..

[bib0044] Mulac A., Hagesaether E., Granas A.G. (2022). Medication dose calculation errors and other numeracy mishaps in hospitals: analysis of the nature and enablers of incident reports. J. Adv. Nurs..

[bib0045] Mulac A., Taxis K., Hagesaether E., Gerd Granas A. (2020). Severe and fatal medication errors in hospitals: findings from the Norwegian incident reporting system. Eur. J. Hosp. Pharm..

[bib0046] NESH. Guidelines for research ethics in the social sciences, humanities, law and theology. At guidelines-for-research-ethics-in-the-social-sciences-and-the-humanities.pdf (forskningsetikk.no) (2016).

[bib0047] Palese A. (2022). The first COVID-19 new graduate nurses generation: findings from an Italian cross-sectional study. BMC Nurs..

[bib0048] Palomino C.L., Ochoa Marín S.C., Restrepo Betancur V., Semenic S. (2023). Impact of the COVID-19 pandemic on the nursing students’ education in a public university in Colombia. Invest. Educ. Enferm..

[bib0049] Piper R. (2018). The mechanistic causes of peripheral intravenous catheter failure based on a parametric computational study. Sci. Rep..

[bib0050] Polit D.F., Beck C.T. (2020).

[bib0051] Ramos-Morcillo A.J., Leal-Costa C., Moral-García J.E., Ruzafa-Martínez M. (2020). Experiences of nursing students during the abrupt change from face-to-face to e-learning education during the first month of confinement due to COVID-19 in Spain. IJERPH.

[bib0052] Ravik M., Bjørk I.T. (2023). Influence of simulation and clinical settings on peripheral vein cannulation skill learning in nursing education: a qualitative study. Int. J. Nurs. Stud. Adv..

[bib0053] Ravik M., Havnes A., Bjørk I.T. (2015). Exploring nursing students’ transfer of peripheral venous cannulation from skills centre to the clinical setting. J. Nurs. Educ. Pract..

[bib0054] Ravik M., Havnes A., Bjørk I.T. (2017). Defining and comparing learning actions in two simulation modalities: students training on a latex arm and each other's arms. J. Clin. Nurs..

[bib0055] Reebals C., Wood T., Markaki A. (2022). Transition to practice for new nurse graduates: barriers and mitigating strategies. West J. Nurs. Res..

[bib0056] Rush K.L., Janke R., Duchscher J.E., Phillips R., Kaur S. (2019). Best practices of formal new graduate transition programs: an integrative review. Int. J. Nurs. Stud..

[bib0057] See E.C.W., Koh S.S.L., Baladram S., Shorey S. (2023). Role transition of newly graduated nurses from nursing students to registered nurses: a qualitative systematic review. Nurs. Educ. Today.

[bib0058] Smith S.M. (2021). Impact of Covid-19 on new graduate nurses’ transition to practice. Loss or gain?. Nurs. Educ..

[bib0059] Soerensen J., Nielsen D.S., Pihl G.T. (2023). It's a hard process – Nursing students’ lived experiences leading to dropping out of their education; a qualitative study. Nurs. Educ. Today.

[bib0060] Stolic S. (2014). Educational strategies aimed at improving student nurse's medication calculation skills: a review of the research literature. Nurs. Educ. Pract..

[bib0061] Stolic S., Ng L., Southern J., Sheridan G. (2022). Medication errors by nursing students on clinical practice: an integrative review. Nurs. Educ. Today.

[bib0062] Sultana N. (2017). An evaluation of drug dosage calculation knowledge and proficiency among newly hired nurses in private tertiary care hospital, Islamabad, Pakistan. J. Clin. Res..

[bib0063] Thirsk L.M. (2022). Effect of online versus in-person clinical experiences on nursing student's competency development: a cross-sectional, quasi-experimental design. Nurs. Educ. Today.

[bib0064] Utli H., Yurt S. (2022). The theory-practice gap in nursing education during the pandemic period from the perspective of stakeholders: a qualitative study. Clin. Exp. Health Sci..

[bib0065] van Rooyen D.R.M., Jordan P.J., ten Ham-Baloyi W., Caka E.M. (2018). A comprehensive literature review of guidelines facilitating transition of newly graduated nurses to professional nurses. Nurs. Educ. Pract..

[bib0066] Visiers-Jiménez L. (2022). Graduating nursing students’ empowerment and related factors: comparative study in six European countries. Healthcare.

[bib0067] WHO (2020).

[bib0068] Woo M.W.J., Newman S.A. (2020). The experience of transition from nursing students to newly graduated registered nurses in Singapore. Int. J. Nurs. Sci..

[bib0069] Yalçınlı S., Akarca F.K., Can Ö., Şener A., Akbinar C. (2019). Factors affecting the first-attempt success rate of intravenous cannulation in older people. J. Clin. Nurs..

[bib0070] Zamanzadeh V., Jasemi M., Valizadeh L., Keogh B., Taleghani F. (2015). Lack of preparation: iranian nurses’ experiences during transition from college to clinical practice. J. Prof. Nurs..

